# Determination of the effects of initial glucose on the production of α-amylase from *Penicillium* sp. under solid-state and submerged fermentation

**DOI:** 10.1080/13102818.2014.901670

**Published:** 2014-06-04

**Authors:** Figen Ertan (İnceoğlu), Bilal Balkan, Zehra Yarkın

**Affiliations:** ^a^ Trakya University, Faculty of Science, Edirne, Turkey; ^b^ Kırklareli University, Vocational College of Health Services, Kırklareli, Turkey

**Keywords:** *Penicillium*, catabolite repression, α-amylase, solid-state fermentation, submerged fermentation

## Abstract

The effects of catabolite repression of initial glucose on the synthesis of α-amylase from *Penicillium chrysogenum* and *Penicillium griseofulvum* were investigated under solid-state fermentation (SSF) and submerged fermentation (SmF) systems. The results obtained from either fermentation were compared with each other. In the SmF system, initial glucose concentration above 10 mg/mL completely repressed the production of α-amylase from *P. chrysogenum* and *P*. *griseofulvum*. However, the repression in the SSF system was not complete, even when the glucose level was raised to 160 mg/g.

## Introduction

A variety of microorganisms, including bacteria, yeasts and filamentous fungi, have been reported to produce amylolytic enzymes.[1–3] α-Amylase (EC 3.2.1.1 α-1,4-glucan-4-glucanohydrolase) is an enzyme which hydrolyses starch. With its endo-acting mechanism, it breaks down α-1,4-glycoside bonds to short-chain oligosaccharides and α-limit dextrin. This enzyme has commercial importance due to production of sugar syrups comprising of glucose, maltose and oligosaccharides. Furthermore, α-amylase is widely used in various fields of industry, including paper, food, pharmaceutical and sugar industries.[4,5] Amylases account for about 30% of the world's enzyme production.[6] Fungal α-amylases can be produced using two main methods, submerged fermentation (SmF) and solid-state fermentation (SSF). Most research has used SmF, which allows control of the medium and of other environmental factors required for the optimum growth of microorganisms.[7] SSF is an economic and easy fermentation technique which does not require complicated machinery and equipment, and control systems. It is highly favourable for industrial process because of lower water and energy demand, higher product yield, lower capital and costs. Besides, SSF has other advantages such as easier control of contamination, owing to the low moisture levels in the fermentation media, absence of foam formation and less downstream processing.[1,8]

Many microbial enzyme syntheses are repressed in the presence of some molecules such as glucose; glycerol and other carbon sources which are known inductor molecules. This is called catabolite repression (CR).[9,10]

CR caused by glucose and other easily metabolizable sugars in the production of amylase and other enzyme by microorganisms developed in SmF is well documented.[2,7,11–13] CR poses serious problems on the economics of SmF for the production of α-amylase and other enzymes. The ability of SSF to significantly minimize CR has been described in the production of different hydrolytic enzymes, including amylolytic systems in some microorganisms.[14,15] It was reported that the production of α-amylase by *Bacillus licheniformis* M27 in SmF was completely inhibited due to repression in medium containing 1% glucose. In contrast, the enzyme production in SSF was 19 550 U/mL in the extract even when the medium contained 15% glucose.[3] Even though *Penicillium* species are reported as a good α-amylase producer.[16–19] So far, there are no reports on the CR of glucose in SSF using *Penicillium* species. The aim of this research was to study the effects of initial glucose on the production of α-amylase from *Penicillium chrysogenum* and *Penicillium griseofulvum* in SSF and SmF.

## Materials and methods

### Fungi

*P. chrysogenum* and *P. griseofulvum* used in this study were isolated from the air in Edirne city, and morphological, physiological and biochemical characterization was performed by Aydogdu and Asan.[20] These fungi were found to be good α-amylase producers and their enzymatic properties were investigated in our previous studies.[17,18] The isolates were maintained on potato dextrose agar (PDA) slants at 4°C.

### Inoculum preparation

A volume of 7 mL of sterile distilled water was transferred to a sporulated (seven-day-old) PDA slant culture. The spores were dislodged using an inoculation needle under aseptic conditions and the suspension, with appropriate dilution, was used as inoculum. One millilitre of the spore suspension (containing about 1 × 10^6^ spores/mL) was used as inoculum in both types of fermentation for the production of α-amylase from *P. chrysogenum* and *P. griseofulvum*.[17,18]

### Enzyme production in submerged fermentation

SmF was carried out in wheat bran extract media prepared by the following procedure. Two hundred grams of wheat bran were boiled in 2000 mL of distilled water for 30 minutes. The solid portion was removed by filtration. The filtrate was collected and added to a mineral salt solution to give final concentrations of ZnSO_4_.7H_2_O (12.4 mg), FeSO_4_ (13.6 mg) and CuSO_4_.7H_2_O (1.60 mg), and was made up to 2000 mL. The pH was adjusted to 5.0. This basal mineral salt (BMS) wheat bran extract medium was poured into 250 mL Erlenmeyer flasks, 45 mL in each and sterilized at 121°C for 15 minutes. Concentrated glucose solutions, which had been sterilized separately, were added to each of these flasks giving the desired glucose concentrations (10, 20, 40, 80 and 160 mg/mL) in a final volume of 50 mL.[13] The flasks were incubated at 30°C in a rotary shaker at 150 r/minute for six days. After the incubation was completed, the fungi were harvested by filtration and the filtrate was used as the crude enzyme preparation and to determine glucose concentrations.

### Enzyme production in solid-state fermentation

Twenty-two and a half grams of dry wheat bran moistened with 25.0 g BMS medium were placed in each 250 mL Erlenmeyer flask and sterilized at 121°C for 30 minutes. Concentrated glucose stock solution, which was sterilized separately, was added to each of these flasks to give glucose concentrations from 10 mg/g to 160 mg/g solid substrate. Care was taken to adjust the final moisture content of each medium to 60% (w/v).[12] SSF media were mixed thoroughly by tapping and incubated at 30°C in the horizontal position for six days, respectively. Fermented substrates were mixed with 0.1 mol/L of acetate buffer (pH 5.0, 50 mL). This mixture was shaken for 1 h at 30°C on a rotary shaker at 220 r/minute. The slurry was squeezed through muslin cloth. The extract was filtered through a Whatman No. 1 filter paper and the filtrate was used as the crude enzyme and to determine glucose concentrations.[21]

### Enzyme assay and determination of remaining glucose

Soluble starch (0.8%) was dissolved in boiling 0.1 mol/L acetate buffer (pH 5.0) and then cooled down to 30°C. Fresh iodine reagent was prepared by diluting 1.0 mL of stock solution (0.5% I_2_ in 5.0% KI) into 500 mL of distilled water containing 5 mL of 5 mol/L HCl. For the assay, 0.1 mL of enzyme solution was placed in a test tube 5 minutes following the addition of 0.2 mL of starch substrate. The reaction was stopped by adding 5.0 mL of iodine reagent. The absorbance was measured at 620 nm against a blank.[22] One unit of α-amylase activity is defined as the amount of enzyme which hydrolyses 0.1 mg of starch in 1 minute at 30°C. The remaining glucose concentration in the media was determined by the o-toluidine method.[23] All the experiments were conducted in triplicate and the mean of the tree with standard deviation (SD) was represented.

## Results and discussion

Many different substrates are used for microbial enzyme production in SSF, but wheat bran is the most preferred substrate.[7,24] That is why, wheat bran was used in our study. The effects of initial glucose on the production of α-amylase from *P. chrysogenum* and *P. griseofulvum* in SSF and SmF were examined.

**Figure 1.**
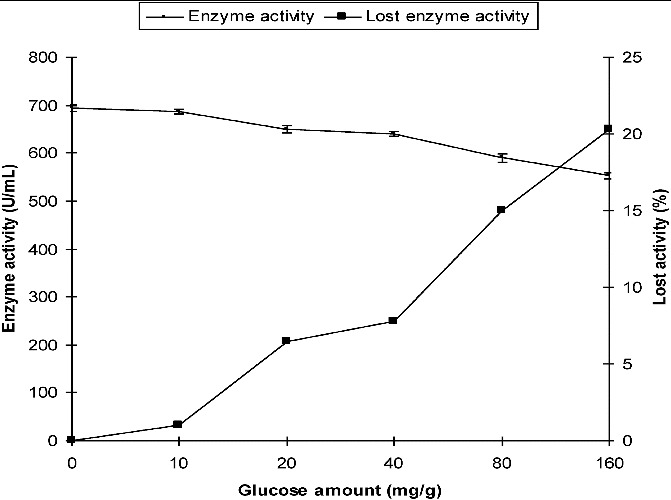
Effect of initial glucose on the production of α-amylase by *P. chrysogenum* in SSF.

The production of α-amylase from *P. chrysogenum* in SSF with different initial concentrations (10, 20, 40, 80 and 160 mg/g) of glucose was compared with that in the control medium which did not contain glucose. The α-amylase activity was 687 U/mL, 649 U/mL and 553 U/mL in SSF in the presence of 10 mg/g, 20 mg/g and 160 mg/g of glucose, respectively. In the control medium, the activity was 694 U/mL. The loss of activity was only 1% at 10 mg/g of initial glucose, whereas the decrease in activity was 6.48% and 20.3% at 20 mg/g and 160 mg/g of initial glucose levels, respectively (Figure 1).

**Figure 2.**
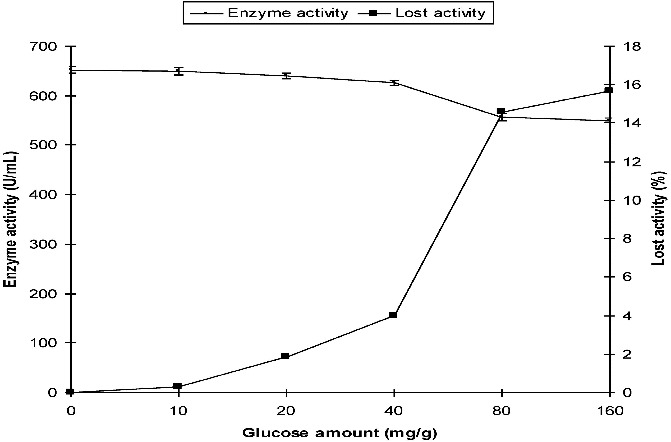
Effect of initial glucose on the production of α-amylase by *P. griseofulvum* in SSF.

By analogy, the production of α-amylase from *P. griseofulvum* in SSF with different initial concentrations (10, 20, 40, 80 and 160 mg/g) of glucose was compared with that in the control medium which did not contain glucose. α-Amylase activity was 652 U/mL in the control medium. In the presence of glucose, the activity was 640 U/mL and 550 U/mL at 20 mg/g and 160 mg/g of initial glucose concentration, respectively. The reduction in the activity was 1.84% and 15.64% at 20 mg/g and 160 mg/g of glucose when compared with those measured in the control medium (Figure 2).

These results show that biosynthesis of α-amylase by both fungi in SSF was insignificantly influenced by the presence of glucose in the media. Complete CR was not observed in the SSF system (Figures 1 and 2).

**Figure 3.**
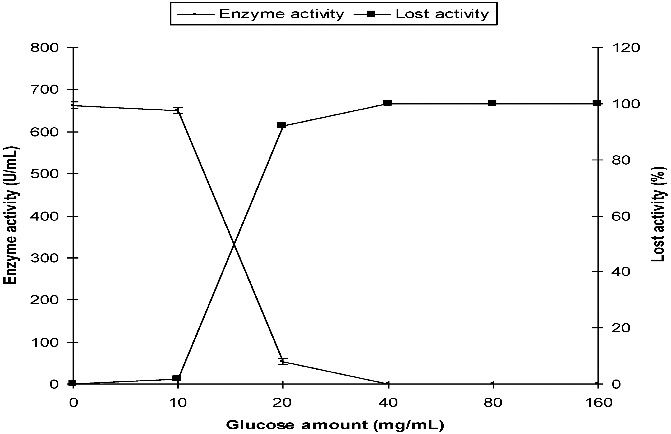
Effect of initial glucose on the production of α-amylase by *P. chrysogenum* in SmF.

*P. chrysogenum* α-amylase activity was 662 U/mL in the control SmF medium. Its activity in the medium containing 20 mg/mL of initial glucose was 53 U/mL. α-Amylase activity was strongly inhibited by higher concentrations of initial glucose, especially at concentrations above 20 mg/mL (Figure 3).

**Figure 4.**
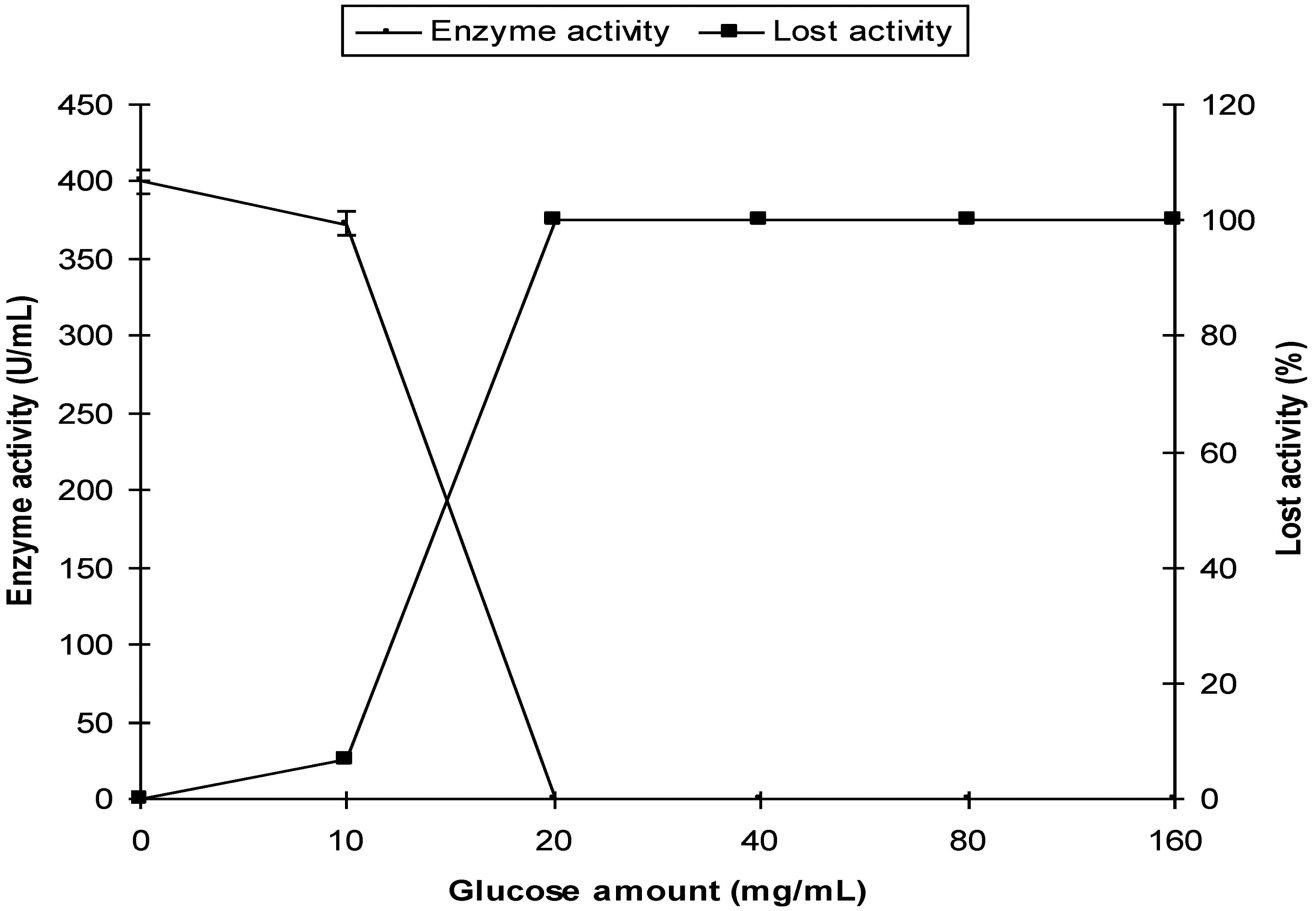
Effect of initial glucose on the production of α-amylase by *P. griseofulvum* in SmF.

The activity of α-amylase produced by *P. griseofulvum* was 400 U/mL in SmF in the absence of glucose (control medium). The activity was 373 U/mL at 10 mg/mL of initial glucose. Enzyme activity, however, was not detected at initial glucose concentrations of 20 mg/mL and above (Figure 4).

**Figure 5.**
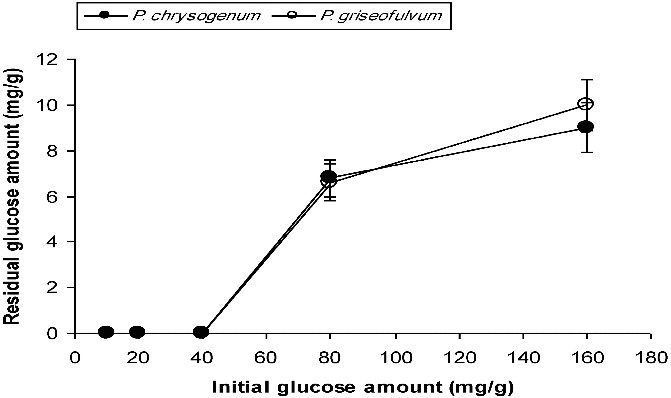
Residual glucose levels in SSF systems at the end of the fermentation process.

**Figure 6.**
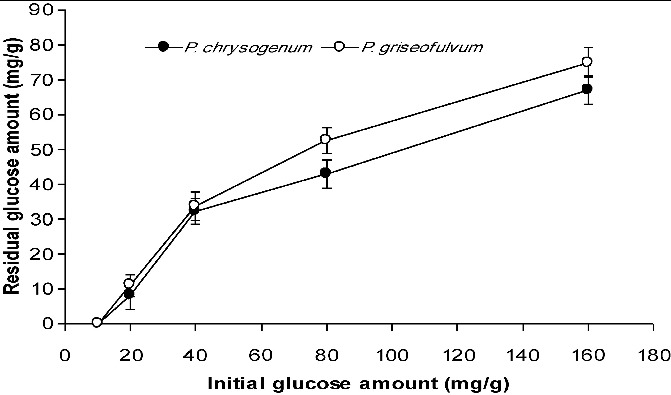
Residual glucose levels in SmF systems at the end of the fermentation process.

For *P. chrysogenum*, the glucose amounts remaining in the medium after six days of SSF were determined as 6.8 mg/g (out of 80 mg/g initially) and 9.0 mg/g (out of 160 mg/g initially). These values for *P. griseofulvum* were determined as 6.6 mg/g and 10 mg/g, respectively, following six days of cultivation (Figure 5). In the case of SmF, the amounts of remaining glucose for *P. chrysogenum* were 8.13 mg/mL, 32.3 mg/mL and 67 mg/mL in the variants with 20 mg/mL, 40 mg/mL and 160 mg/mL of initial glucose, respectively. For *P. griseofulvum*, the remaining glucose levels were determined as 11 mg/mL, 33.72 mg/mL and 75 mg/mL in the variants with 20 mg/mL, 40 mg/mL and 160 mg/mL of initial glucose concentrations, respectively (Figure 6).

High α-amylase activities were found in all glucose concentrations tested in SSF when compared with SmF. These results indicated that CR was minimized in the SSF system when compared with SmF. Similar results were reported for bacterial and fungal cultures.[10] This may be due to the lower water activity and absence of medium agitation in SSF, which considerably reduces the diffusion process in the medium. Thus, the SSF system with its reduced level of CR is more cost effective than SmF.[13] Furthermore, Maldonado and Saad [25] studied the production of pectinesterase and polygalacturonase by *Aspergillus niger* in SmF and SSF systems and found that the membranes of cells from the SSF showed increased levels of C18:1, C16:0 and C18:0 fatty acids. This observation may be taken as an indication of the changes of permeability of fungi grown by the SSF technique.[10]

The losses of activity for both fungi were determined to be ∼20% also at high initial glucose concentrations under SSF. In the case of SmF, however, the observed losses for both fungi were 100% at relatively low initial glucose levels. These results are in accordance with previous studies demonstrating that SSF systems using wheat bran as a substrate are resistant to CR. The use of an absorbent support such as wheat bran helps the SSF system to become more resistant to CR.[19]

The amounts of remaining glucose in SmF were higher than those in SSF (Figures 5 and 6). The amounts of residual glucose in both processes were directly proportional to the loss of activity. Taken together, the obtained results indicate that SSF is a more economical than SmF for α-amylase production by *Penicillium* sp.

## Conclusions

In this study, it was observed that α-amylase activity in *Penicillium* sp. was slightly repressed by initial glucose under SSF with wheat bran as a substrate. However, initial glucose caused strong repression of α-amylase synthesis in *Penicillium* sp. under SmF. Therefore, the SSF system could be suggested as an economical and successful process for α-amylase production by *Penicillium* sp. in the absence or presence of glucose. 
